# Nonmuscle myosin 2 proteins encoded by *Myh9*,* Myh10*, and *Myh14* are uniquely distributed in the tubular segments of murine kidney

**DOI:** 10.14814/phy2.13513

**Published:** 2017-12-06

**Authors:** Karla L. Otterpohl, Ryan G. Hart, Claire Evans, Kameswaran Surendran, Indra Chandrasekar

**Affiliations:** ^1^ Enabling Technologies Group ‐ Sanford Research Sioux Falls South Dakota USA; ^2^ Molecular Pathology Core Sanford Research Sioux Falls South Dakota USA; ^3^ Pediatrics and Rare Diseases Group ‐ Sanford Research Sioux Falls South Dakota USA; ^4^ Department of Pediatrics USD Sanford School of Medicine Sioux Falls South Dakota USA

**Keywords:** Nonmuscle myosin 2, renal epithelium expression

## Abstract

The diverse epithelial cell types of the kidneys are segregated into nephron segments and the collecting ducts in order to endow each tubular segment with unique functions. The rich diversity of the epithelial cell types is highlighted by the unique membrane channels and receptors expressed within each nephron segment. Our previous work identified a critical role for Myh9 and Myh10 in mammalian endocytosis. Here, we examined the expression patterns of Nonmuscle myosin 2 (NM2) heavy chains encoded by *Myh9*,* Myh10*, and *Myh14* in mouse kidneys as these genes may confer unique nephron segment‐specific membrane transport properties. Interestingly, we found that each segment of the renal tubules predominately expressed only two of the three NM2 isoforms, with isoform‐specific subcellular localization, and different levels of expression within a nephron segment. Additionally, we identify Myh14 to be restricted to the intercalated cells and Myh10 to be restricted to the principal cells within the collecting ducts and connecting segments. We speculate that the distinct expression pattern of the NM2 proteins likely reflects the diversity of the intracellular trafficking machinery present within the different renal tubular epithelial segments.

## Introduction

Nephrons are the structural and functional units of the kidney that filter blood, and are each composed of a glomerulus, the filtration unit, and a series of unique tubular segments that refine the filtrate. Refinement of the renal filtrate along the tubular segment is carried out by reabsorption of water, ions, glucose, amino acids, vitamins and other molecules via endocytosis, diffusion, and active transport. The renal tubules can be classified into four segments based on the expression of receptor molecules, transporters, and ion/water channels that determine its function. The first segment which connects to the Bowman's space of the glomerulus is the proximal tubule followed by the loop of Henle, the distal tubule, and the collecting duct segment. The proximal segment is characterized by expression of the apical receptors megalin and cubilin which mediate endocytosis of proteins (Christensen et al. 2012; De et al. 2014) such as albumin (Cui et al. 1996), vitamin carrier proteins (Christensen et al. 1999; Nykjaer et al. 1999), lipoproteins (Stefansson et al. 1995), enzymes (Christensen et al. 2007), microglobulins (Leheste et al. 1999), and certain drugs (Moestrup et al. 1995). Proximal tubules are therefore responsible for most of the nutrient uptake from the filtrate. Furthermore, almost 90% of the filtered bicarbonate and about 60–70% sodium chloride are reabsorbed along the proximal tubules (Curthoys and Moe 2014). The loop of Henle is involved in reabsorption of sodium and chloride ions from the filtrate. The filtrate also gets diluted along the thick ascending limb (TAL) of this segment, since it is impermeable to water while actively reabsorbing the ions from the filtrate (Pannabecker 2012; Mount 2014). The regulation of salt transport along the TAL segment involves coordinated functions of apical and basolateral membrane‐associated ion transporters and channels. Ion transporters like NKCC2 localize to the apical membrane of TAL and facilitate reabsorption of sodium and chloride ions (Gamba and Friedman 2009; Mount 2014). The potassium ions entering through the apical membrane are recycled back to the lumen via the inward rectifier potassium channel, ROMK (Welling and Ho 2009), while the sodium and chloride ions leave the cell through the basolateral Na^+^ K^+^ ATPase (Feraille and Doucet 2001) and the chloride channels (Reeves et al. 2001). Apical membrane localization of some of the ion transporters like NHE3 and NaPi2 in renal proximal tubules are regulated by associations with scaffolding proteins and the actin cytoskeleton (Weinman et al. 2005; Blaine et al. 2009). The distal tubular segment also regulates potassium, calcium, and sodium reabsorption through ion channels and transporters (Subramanya and Ellison 2014). The final segment, the collecting duct, maintains the body's electrolyte and fluid balance by fine‐tuning the amount of water and electrolytes excreted or reabsorbed (Pearce et al. 2015; Roy et al. 2015). The localization of Aquaporin‐2 (water channel) and the ENaC (sodium channel) on the apical membrane of the collecting duct epithelium are regulated by arginine vasopressin and aldosterone, respectively (Pearce et al. 2015). Thus, receptor‐mediated endocytosis and regulated membrane localization of transporters along the nephrons are essential for normal kidney function and maintenance of blood homeostasis.

Diseases affecting the kidney are often undetectable at early stages due to the ability of the kidneys to regulate and maintain normal serum homeostasis even with loss of some nephrons. Unfortunately, this late detection of disease hampers our understanding of disease etiology, pathophysiology, and progression. The advent of genomic sequencing has led to the identification of genetic determinants in both sporadic and hereditary kidney disease (Seri et al. 2003; Chadha and Alon 2009; Freedman et al. 2009; Behar et al. 2010). Interestingly, approximately a third of these mutations are found in known transport and trafficking genes including those responsible for Dents disease, Lowe's Syndrome, Bartter syndrome, Gitelman Syndrome, and Liddle Syndrome (Chadha and Alon 2009; Devuyst and Thakker 2010).

Although genetic sequencing and genome‐wide association studies can identify genes contributing to and increasing the risk of kidney disease, the mechanistic roles of the proteins encoded by these genes are still unclear. One such gene is *MYH9* that encodes for the heavy chain of Nonmuscle myosin 2 (NM2) isoform A. *MYH9* mutations have been associated with several human syndromes, now grouped under the terminology of *MYH9*‐related disorders, which involve hearing loss, thrombocytopenia, cataracts, and kidney disease (Heath et al. 2001; Arrondel et al. 2002; Mhatre et al. 2003; Seri et al. 2003; Sekine et al. 2010). NM2 is a complex hexameric protein composed of two heavy chains and four light chains that play an essential role in cell polarization, adhesion, and migration (Vicente‐Manzanares et al. 2009). The N‐terminal globular head domain of NM2 binds to actin and ATP. The C‐terminal filament assembly domain interacts with another myosin hexamer to form the four‐headed bipolar filament. Bundles of bipolar filaments organized on polarized actin filaments undergo progressive movement through the Mg^2+^ ATPase activity which results in generation of mechanical force and tension within cells (Vicente‐Manzanares et al. 2009). The three isoforms of NM2 are NM2A, NM2B, and NM2C whose heavy chains are encoded by genes *MYH9*,* MYH10*, and *MYH14*, respectively. Functional studies of the three NM2 isoforms in mice have elucidated both unique and redundant functions for Myh9 and Myh10 (Wang et al. 2011). Currently, the importance of Myh14 is unknown, as global knockout of *Myh14* in mice has no obvious phenotype (Ma et al. 2010). Recently, we have established a novel role for Myh9 and Myh10 during mammalian endocytosis in neurons and fibroblasts (Chandrasekar et al. 2013, 2014). Recent reports have shown that Myh9 plays a critical role in Golgi trafficking (Miserey‐Lenkei et al. 2010). Based on these findings, we hypothesize that NM2 isoforms play an important role in intracellular transport in the specialized renal tubular epithelial cells. We hypothesize that NM2 isoforms might participate and selectively regulate renal epithelial transport either by controlling the apical membrane dynamics or by directly interacting with membrane or vesicle associated proteins and facilitating the organelle trafficking pathways. All three NM2 gene products are expressed in the mammalian kidneys as determined by immunoblotting (Golomb et al. 2004). However, renal expression pattern analysis of NM2 proteins has predominately been focused on the glomeruli. These studies have localized both MYH9 and MYH10 proteins specifically to the podocytes and mesangial cells within renal glomeruli (Arrondel et al. 2002; Miura et al. 2013). Several groups have used murine models of *Myh9* deletion in the glomerulus to uncover the mechanism of kidney disease in patients with *MYH9*‐related disorders; however, these models have failed to provide clear evidence of disease development (Johnstone et al. 2011, 2013). As a first step to understand the possible roles of NM2 proteins in the mature renal tubular epithelium, we characterized the expression of Myh9, Myh10, and Myh14 in the renal tubules of adult mice. Our analysis of NM2 expression in the adult kidney revealed that Myh9 and Myh10 are expressed in distinct regions of the renal tubules; whereas, Myh14 is predominantly expressed within the same segments as Myh10. The distinct expression pattern of the NM2 proteins likely reflects the diversity of the intracellular trafficking machinery present within the different renal tubular epithelial segments.

## Materials and Methods

### Animal care

All mice used were housed in facilities at the Sanford Health Research Center accredited by the American Association for Accreditation of Laboratory Animal Care. All procedures involving live mice were approved by the Sanford Health Research Center Institutional Animal Care and Use Committee.

### Immunohistochemistry for NM2 location in renal sections

Kidneys were collected from mice with a mixed genetic background (C57BL/6J, 129sv, DBA and FVB/N) at 12 weeks of age and fixed in Bouin's fixative prior to paraffin embedding. Kidneys were also collected from 12‐week‐old mice perfused with 4% Paraformaldehyde (Electron Microscopy Sciences) and cacodylate buffer (Electron Microscopy Sciences) and placed in Tissue‐Tek OTC Compound (27050, Ted Pella, INC.) and frozen for sectioning. Following rehydration of paraffin‐embedded tissues, the sections were stained following a modified protocol from Surendran et al. (2010). Briefly, Triology (Cell Marque) was used for antigen retrieval and sections were blocked for 15 min using a modified blocking solution (1% bovine serum albumin (Sigma‐Aldrich), 0.2% milk, 4% Fish gelatin (Sigma‐Aldrich), and 0.3% Triton X‐100 (Sigma‐Aldrich) in PBS). The polyclonal rabbit antibodies against the NM2 isoforms were developed using a synthetic polypeptide antigen designed after the C‐terminal (tail segment) amino acid sequences that are unique to each isoform (Maupin et al. 1994; Phillips et al. 1995; Golomb et al. 2004). These antibodies have been widely used in multiple publications for immunohistochemistry, immunofluorescence staining in cells, and immunoblots and have been proven to have excellent isoform specificity without any cross reactivity. We have used these antibodies in MEF's isolated from *Myh10* KO mice and our isoform‐specific knockdown cells successfully that provides further evidence for its specificity and no cross reactivity (Chandrasekar et al. 2014). Primary antibodies (1:500 dilution Myh9, 909801, Biolegend; 1:500 dilution Myh10, 909901, Biolegend; 1:500 dilution Myh14, 919201, Biolegend; 1:100 dilution Megalin, ab184676, Abcam; 1:200 dilution Uromodulin, PA5‐47706, Thermo Scientific; 1:1000 dilution Calbindin D‐28K, NBP2‐50028, Novus Biologicals; 1:200 dilution Aquaporin‐2, sc9882, Santa Cruz Biotechnology; 1:5000 dilution Aquaporin‐2, AQP‐002, Alomone Labs; 1:100 V‐ATPase B1 (Atp6v1b1), sc‐21206, Santa Cruz Biotechnology) were incubated for 1 hour at room temperature. Secondary antibodies (1:500 dilution CY3‐conjugated donkey anti‐rabbit, 711‐165‐152, Jackson Immuno Research; 1:500 Alexa‐Fluor 488‐conjugated donkey anti‐goat, 705‐545‐147, Jackson Immuno Research; 1:500 dilution Alexa‐Fluor 488‐conjugated donkey anti‐chicken, 703‐545‐155, Jackson Immuno Research; 1:500 dilution CY5‐conjugated donkey anti‐mouse, 715‐175‐150, Jackson Immuno Research; 1:500 dilution Alexa‐Fluor 488‐conjugated donkey anti‐rabbit, 711‐545‐152, Jackson Immuno Research; 1:500 dilution DyLight 549‐conjugated horse anti‐mouse, DI‐2549, Vector Laboratories; 1:500 Alexa‐Fluor 488‐conjugated donkey anti‐mouse, 715‐545‐150, Jackson Immuno Research; 1:500 Alexa‐Fluor 488‐conjugated donkey anti‐sheep, A11015, Invitrogen; Alexa‐Fluor 647‐conjugated donkey anti‐sheep, 713‐605‐147, Jackson Immuno Research; 1:250 CY3‐conjugated donkey anit‐goat, 705‐165‐147, Jackson Immuno Research; 1:250 Alexa‐Fluor 647 donkey anti‐rabbit, A31547, Life Technologies) were incubated for 1 h at room temperature following three washes with PBS. Frozen sections were blocked and incubated following the same protocol as the paraffin‐embedded samples (Myh9: 1:250 dilution; Myh10: 1:250 dilution; or Myh14: 1:250 dilution; 1:500 dilution Alexa‐Fluor 488‐Phalloidin, A12379, Invitrogen; 1:500 dilution Cy3‐conjugated donkey anti‐rabbit). Coverslips were mounted using the Vector Shields fluorescence mounting media with DAPI (H‐1200, Vector Laboratories).

### Analysis of NM2 localization in the renal tubules

Sections were imaged using the Nikon A1 confocal microscope and brightness and color adjusted using the ImageJ software. To determine if NM2 was expressed in each of the four segments of the renal tubules, 100 tubules were scored for coexpression of NM2 with one of four markers. Megalin was used for the proximal segment, uromodulin for the thick ascending limb of the loop of Henle, calbindin D‐28k for the distal segment, and aquaporin‐2 for the collecting duct. Atp6v1b1 was used to identify intercalated cells.

## Results

### Expression and localization pattern of NM2 isoforms in the proximal tubule

**Figure 1:**
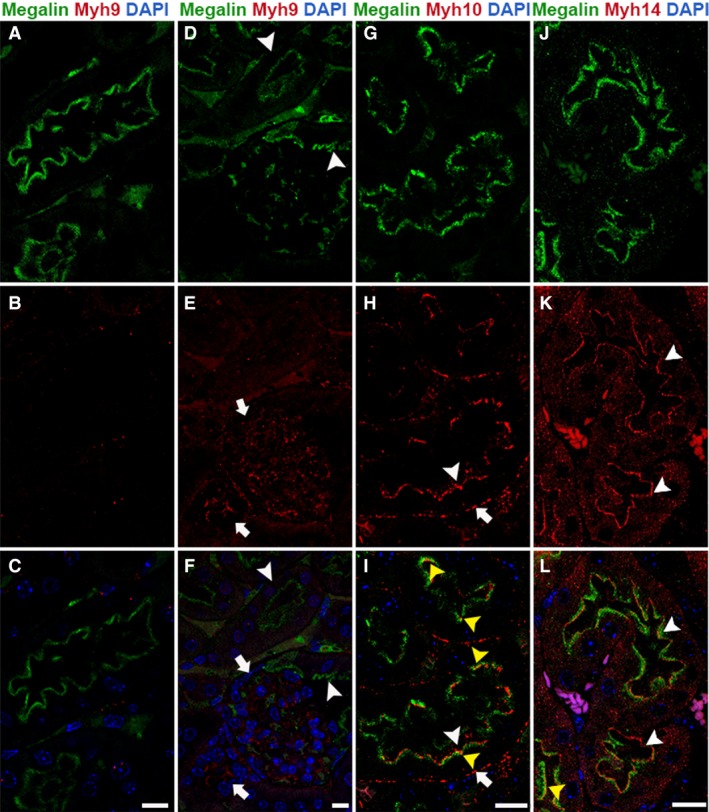
Myh10 and Myh14 are the NM2 isoforms expressed in the proximal tubular segment. Confocal fluorescence microscopy of the adult mouse kidney sections stained for megalin (proximal tubule marker) and one of the NM2 isoforms revealed that Myh10 and Myh14 are the prominent isoforms of NM2 in the proximal tubules. Myh9 (red) is not expressed in the megalin (green)‐positive proximal tubules (arrowheads, A–F); however, Myh9 expression is present in the adjacent tubules and the glomerulus (arrows, D–F). Myh10 localized to apical (arrowhead) and basolateral (arrow) membranes in the megalin‐positive proximal tubular segments (G–I). Myh14 was expressed in the megalin‐positive tubules on the apical membrane (arrowhead); punctate structures were also observed on the basolateral membrane and within the epithelial cells (J–L). The majority of Myh10 and Myh14 does not colocalize with megalin on the apical membrane, and appear to be above the megalin staining on the membrane. However, there are a few regions (microdomains) on the apical membrane (yellow arrowhead, I&L) where we observe partial colocalization. Sections were mounted with vectashield containing DAPI to stain the nuclei (blue; C, F, I & L). Scale bar = 10 *μ*m.

The proximal tubule of the nephron connects the Bowman's capsule to the loop of Henle and reabsorbs the majority of the filtrate. The apical membrane of the proximal tubular epithelial cells has a distinct group of microvilli‐like structures named brush borders. Brush borders aid in reabsorption of proteins, salts, and minerals through the various receptors as well as act as a flow sensor in the lumen. Megalin and cubilin are the major endocytic receptors of the proximal tubules (Christensen et al. 2012; De et al. 2014). We analyzed the expression and localization pattern of NM2 proteins in the proximal tubular segment by immunofluorescence staining utilizing an anti‐megalin antibody to identify the proximal tubular segments. Confocal microscopy of the stained mouse kidney tissues indicates that only two of the NM2 proteins are expressed in megalin‐positive proximal tubular segments (Fig. 1). To quantify the expression pattern of NM2 proteins in adult mouse renal proximal tubules, we analyzed 100 megalin‐positive tubular segments from at least three different kidney sections. Myh10 and Myh14 are the major isoforms expressed in proximal tubules with 70% and 87% of megalin‐positive tubules expressing these isoforms, respectively. Myh9 was not expressed in the megalin‐positive proximal tubules (Fig. 1A–F) as evident by high levels of Myh9 staining observed in the glomerulus in the same sections (Fig. 1D–F). The localization pattern of the NM2 isoforms within the proximal tubular epithelial cells also varied. Myh14 was predominately localized along with megalin on the apical membrane of the tubules (Fig. 1J–L). Intracellular punctate structures of Myh14 were also visible within the epithelial cells. Myh10 was localized to both the apical (arrowhead) and basolateral (arrow) membranes of the proximal tubules, and additionally intracellular punctate structures were also observed in a few cells (Fig. 1G–I). The majority of Myh10 and Myh14 does not colocalize with megalin on the apical membrane. The megalin staining appears to be subapical to the Myh10 and Myh14 on the membrane. However, there are a few regions (microdomains) on the apical membrane (Fig. 1I and L) where we observe partial colocalization.

### Expression and localization pattern of NM2 isoforms in the loop of Henle

**Figure 2:**
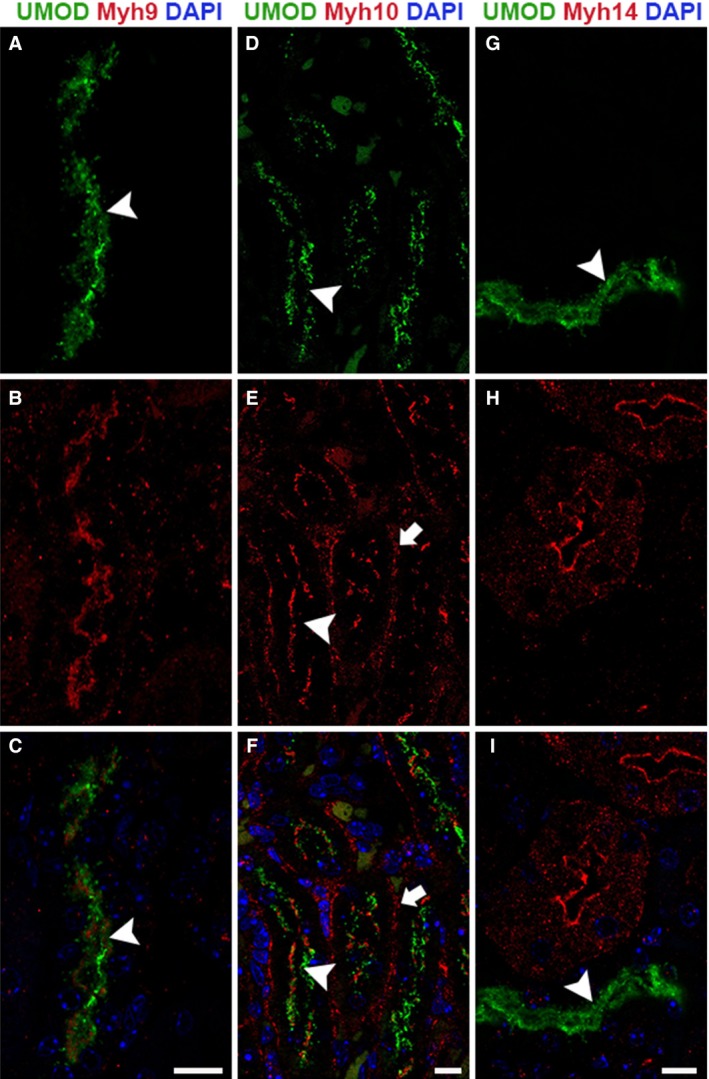
Myh9 and Myh10 are the NM2 proteins expressed in the thick ascending limb of the loop of Henle segment. Confocal fluorescence microscopy of the adult mouse kidney sections stained for uromodulin (TAL marker) along with the NM2 proteins individually revealed that Myh9 and Myh10 are the major NM2 proteins in TAL segment of the Loop of Henle tubules. Myh9 (red) is highly expressed in the majority of the uromodulin (green) TAL tubules, with strong localization on the apical membrane (A–C, arrowhead). Myh10 localized to the apical (arrowhead) and basolateral membranes (arrow) in most of the uromodulin‐positive TAL segments (D–F). Myh14 was not expressed in the TAL tubules, although several of the adjacent uromodulin‐negative tubules expressed Myh14 (G–I). Sections were mounted with vectashield containing DAPI to stain the nucleus (blue; C, F & I). Scale bar = 10 *μ*m.

The loop of Henle connects the proximal tubular segments to the distal regions and consists of thin descending limb, thin ascending limb, and thick ascending limb (TAL). The epithelial cells express cotransporters, receptors, and channels for sodium, potassium, chloride, and calcium and are essential for regulated ion transport along the nephron (Pannabecker 2012; Mount 2014). TAL epithelial cells express a membrane‐bound glycosyl‐phosphatidylinositol (GPI)‐anchored protein, uromodulin, also known as Tamm‐Horsfall glycoprotein (Devuyst et al. 2017). Uromodulin can be utilized as a marker to visualize and identify the thick ascending limb tubules. We performed immunofluorescence staining of adult mouse kidney sections using anti‐uromodulin antibody in combination with antibody against one of the three NM2 proteins. Confocal microscopy of stained kidney sections revealed that Myh9 and Myh10 are the major isoforms expressed in uromodulin‐positive thick ascending limb tubules. Myh9 and Myh10 colocalized with uromodulin in 95% and 98% of thick ascending limb tubules, respectively. The Myh14 isoform was not expressed in the TAL tubules (Fig. 2G–I). Myh9 localized to the apical membrane along with uromodulin (Fig. 2A–C). Myh10 localized to the apical and basolateral membrane in the uromodulin‐positive TAL segments (Fig. 2D–F).

### Expression and localization pattern of NM2 isoforms in distal tubules

**Figure 3:**
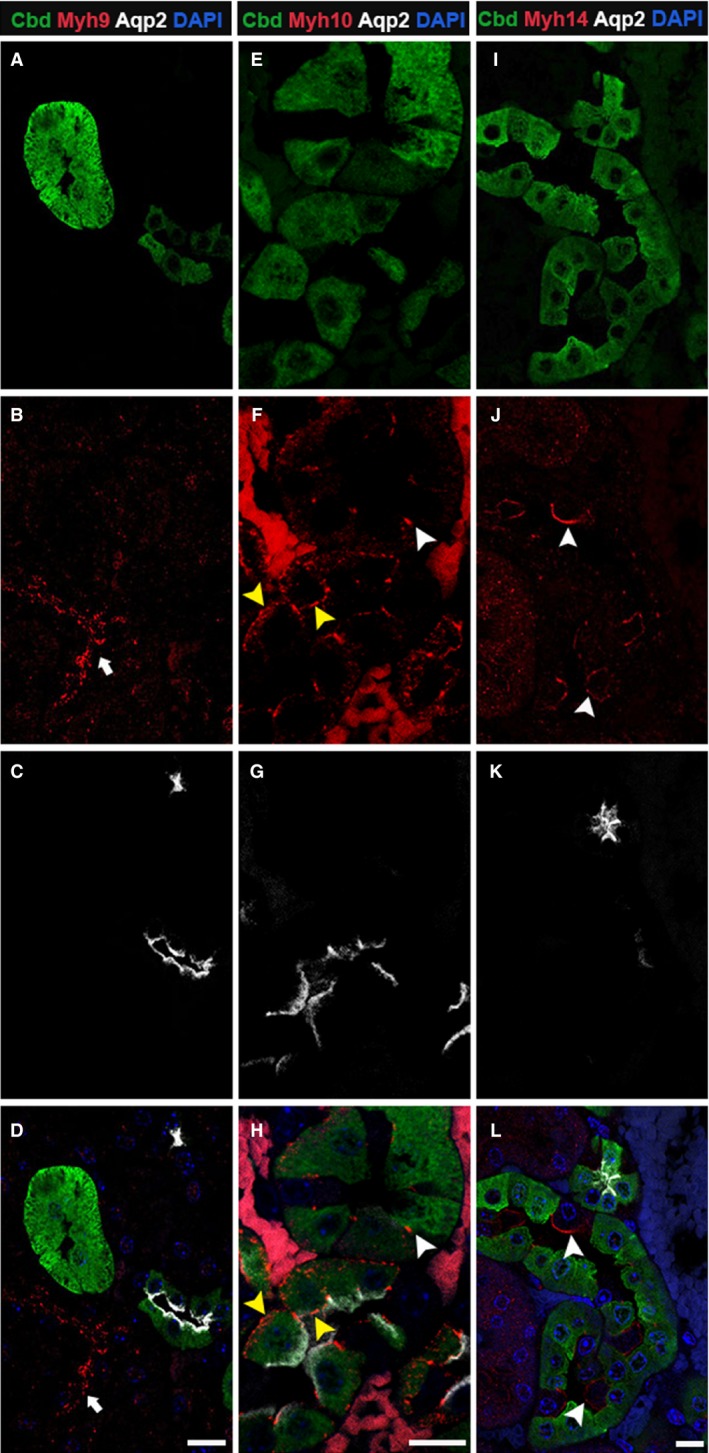
Myh10 and Myh14 are the NM2 isoforms expressed in the distal and connecting renal tubules. Adult kidney sections were stained for calbindin‐D28K, Aqp2 and one of the NM2 isoforms. Myh9 (A–D) was not expressed in tubules positive for calbindin‐D28K and Aqp2; although adjacent tubules were positive for Myh9 (arrow). Myh10 (E–H) was expressed at low levels at the cell–cell junctions in calbindin‐D28K only positive tubules (white arrowhead), and in tubules positive for both calbindin‐D28K and Aqp2, Myh10 localized to cell–cell junctions, apical, and basolateral membrane (F & H, yellow arrowhead). Myh14 (I–L) was expressed on the apical membrane of calbindin‐D28K and Aqp2 null cells (arrowheads) in the distal tubular segments. Sections were mounted with vectashield containing DAPI to stain the nucleus (blue; D, H, and L). Scale bar = 10 *μ*m.

The distal convoluted tubule (DCT) functions in ion transport through the utilization of cotransporters, channels, and mineralocorticoid receptors (Subramanya and Ellison 2014). DCT segments within the adult mouse kidney were identified by calbindin‐D28K expression. However, calbindin‐D28K can also be found in the connecting tubule (CNT), the region of tubule that connects the DCT to the collecting duct. To distinguish between the DCT and CNT, we utilized Aquaporin‐2 (Aqp2), a water channel protein that localizes to the CNT as well as the collecting duct. Therefore, tubules expressing only calbindin‐D28K are DCT, whereas tubules expressing both calbindin‐D28K and Aqp2 are CNT. Analysis of the stained sections indicates that the Myh9 protein was not expressed in the DCT or CNT segments (Fig. 3B). However, both Myh10 and Myh14 were expressed in the DCT and CNT. The Myh10 isoform was expressed at low levels, but specifically localized to the cell–cell junctions in the tubules positive for calbindin‐D28K only (Fig. 3F and H). In the connecting tubular segment, Myh10 was expressed and specifically localized to both the apical and basolateral membranes as well as the cell–cell junctions (Fig. 3F and H). Quantification indicated that 84% of calbindin‐D28K‐positive DCT and 97% of the CNT segments expressed Myh10. The Myh14 isoform was expressed in 88% of calbindin‐D28K‐positive and 80% of calbindin‐D28K and Aqp2 double‐positive tubular segment. Myh14 was not expressed in cells that were positive for calbindin‐D28K and/or Aqp2 within the distal tubule and CNT segments. Instead, Myh14 localized to the apical membranes of cells that were adjacent to the calbindin‐D28K‐ and Aqp2‐positive cells indicating that Myh14 is localized to intercalated cell types in these tubules (Fig. 3I–L).

**Figure 4:**
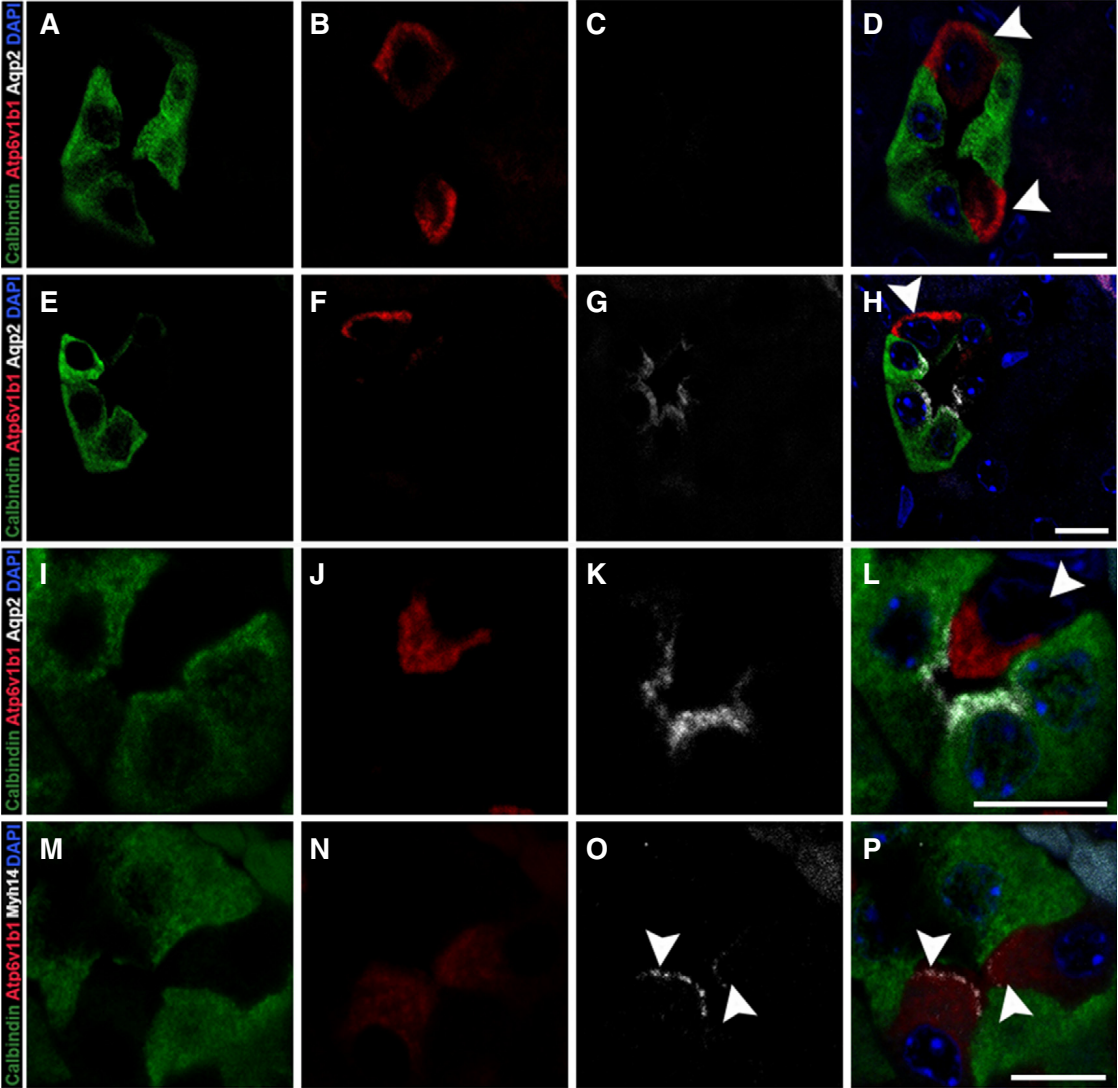
Myh14 localizes to apical membranes of Atp6v1b1‐positive intercalated cells in the distal tubule. Adult kidney sections were stained for calbindin‐D28K, Atp6v1b1, Aqp2, and Myh14 to identify intercalated cells within the distal and connecting tubule segments as well as to confirm Myh14 localization to the intercalated cell type. Cells with positive staining for Atp6v1b1 (red) can be found in tubule segments positive for calbindin‐D28K only (A–D) as well as in tubules positive for both calbindin‐D28K and Aqp2 (E–H); in both cases Atp6v1b1 staining is observed along the basolateral membrane indicating *β*‐ intercalated cell type (arrowhead, D&H). We also observed Atp6v1b1 staining in majority of the tubules along the apical membrane indicating the *α*‐ intercalated cell type (arrowhead, I–L). Myh14 localized to the apical membrane of the intercalated cell type in the distal tubule (arrowhead, M–P). Sections were mounted with vectashield containing DAPI to stain the nucleus (blue; D, H, L and P). Scale bar = 10 *μ*m.

In order to confirm the identity of the Myh14‐positive cell type in the distal tubular segment, we performed immunostaining of adult mouse kidney tissues with Atp6v1b1, a subunit of vacuolar‐ATPase that is involved in organelle acidification, which serves as a marker for intercalated cells. We costained these tissue sections with calbindin‐D28K and Aqp2 to differentiate between DCT and CNT segments. In all of the calbindin‐D28K only positive tubule segments, only a few cells expressed Atp6v1b1 and these were calbindin‐D28K negative, confirming the presence of intercalated cells in the distal tubules (Fig. 4A–D). Intercalated cells were also found in the CNT segment (Fig. 4E–H and Fig. 4I–L). We observed both *α*‐ and *β*‐ intercalated cells in these segments that we could differentiate based on the apical (Fig. 4I–L) or the basolateral (Fig. 4A–H) localization of Atp6v1b1. Immunostaining for Myh14 and Atp6v1b1 together showed that Myh14 is only expressed in the intercalated cells and localizes to the apical membrane (Fig. 4M–P).

### Expression and localization pattern of NM2 isoforms in collecting duct

**Figure 5:**
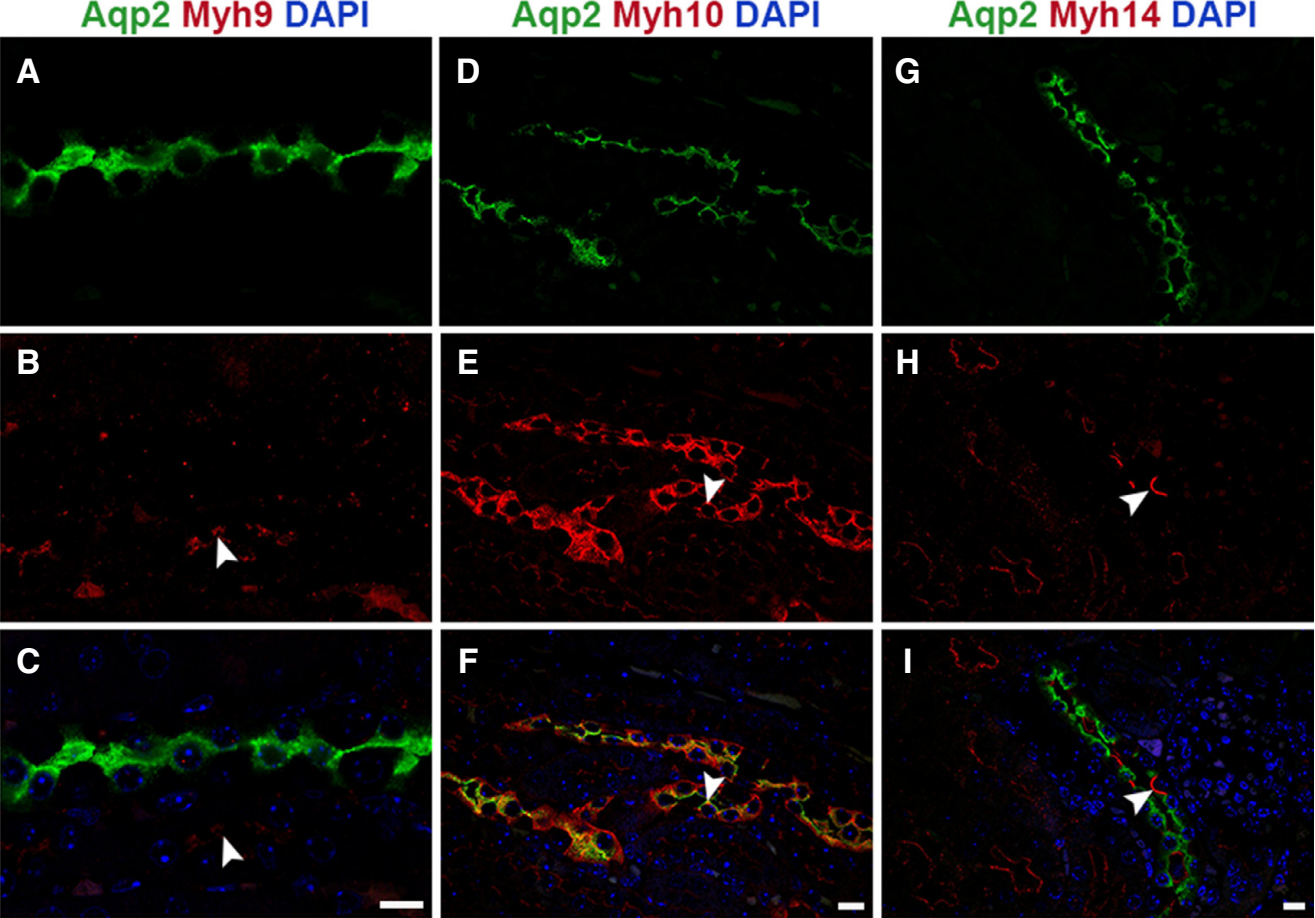
Myh10 and Myh14 isoforms show unique expression and localization pattern in the collecting duct. Kidney sections were stained with Aqp2 to identify the collecting duct segment and costained for the NM2 isoforms. Analysis showed that Myh9 (A–C) was not present in the collecting duct, Myh9 staining was present in the adjacent tubule (arrowhead). Myh10 (D–F) was the most abundant of the isoforms expressed in the collecting duct and localized to apical (arrowhead) and basolateral membrane as well as cell–cell adhesions. Myh14 (G–I) expression in the collecting duct was limited to the apical membranes (arrowhead) of cells not expressing Aqp2 (intercalated cells). Sections were mounted with vectashield containing DAPI to stain the nucleus (blue; C, F, and I). Scale bar = 10 *μ*m.

The collecting duct extends from the CNT to the papilla and plays an important role in water reabsorption and pH regulation (Pearce et al. 2015; Roy et al. 2015). We utilized aquaporin‐2 (Aqp2), a vasopressin‐regulated water channel as a marker to identify collecting ducts. We performed immunofluorescence staining of mouse kidney sections with antibodies against Aqp2 and one of the three NM2 isoforms and analyzed using confocal microscopy. Among the three NM2 isoforms, we found that Myh10 and Myh14 were expressed in nearly all Aqp2‐positive tubule segments (100% and 98%, respectively), whereas Myh9 was not expressed in any of the Aqp2‐positive collecting duct segments (Fig. 5A–C). The Myh10 protein was observed in apical and basolateral membranes as well as cell–cell adhesions of only the Aqp2‐positive cells (Fig. 5D–F). In addition, we also observed intracellular expression of Myh10 in some collecting duct segments. Intracellular expression was observed in all the medullary collecting ducts and occasionally in the cortical regions (29%). Myh14 localized to the apical membrane of the cells next to the Aqp2‐positive cells (Fig. 5G–I), which suggests that Myh14 is expressed in intercalated cells.

### Expression and localization pattern of NM2 isoforms and actin filaments in mouse renal tubules

**Figure 6:**
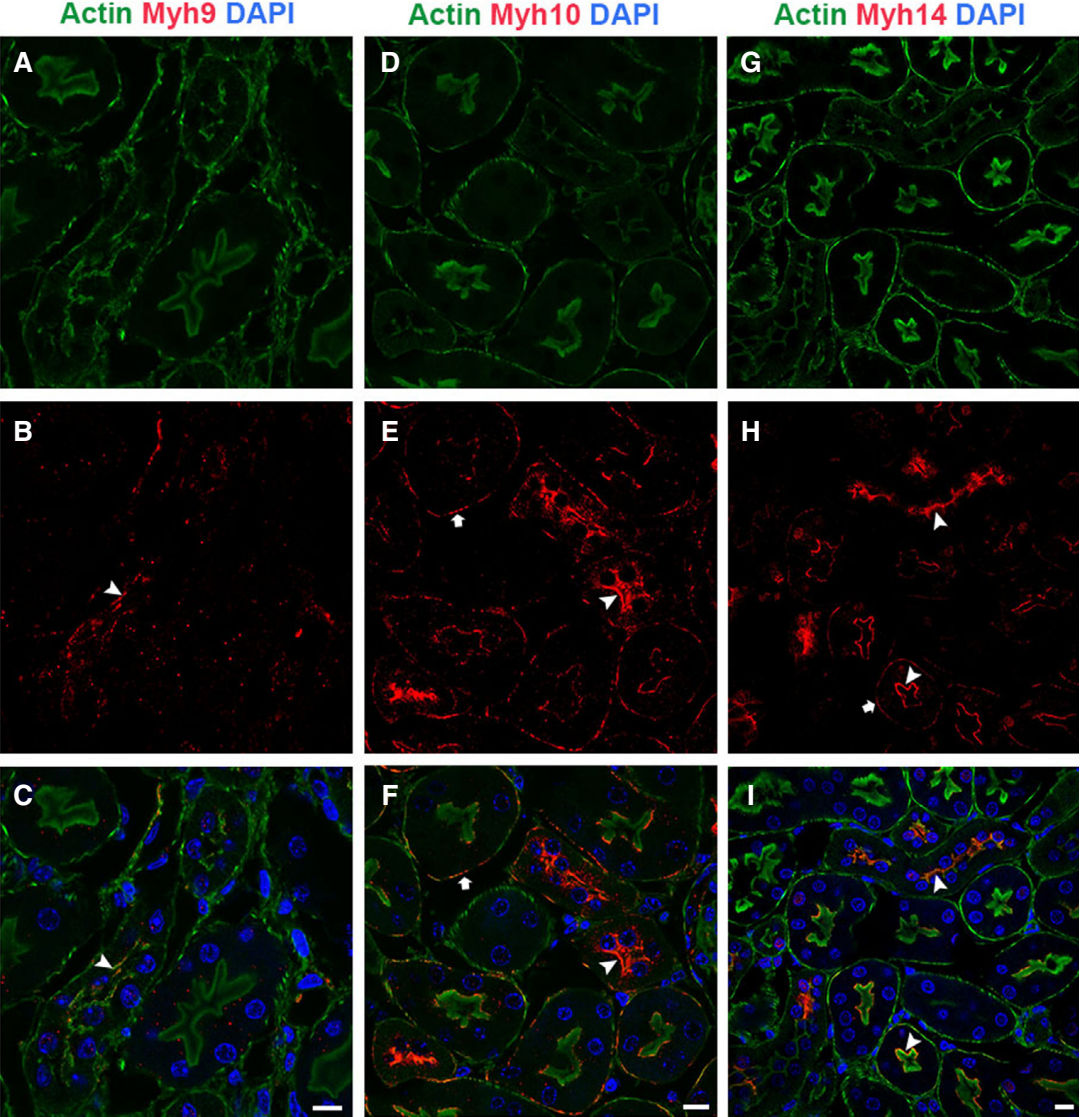
NM2 isoforms have selective expression and localization pattern with actin filaments in the renal tubules. Confocal analysis of sections stained for phalloidin and NM2 isoforms showed that NM2 isoforms did not colocalize with all the filamentous actin structures in the tubules. Myh9 colocalized with actin filaments on the apical membrane (A–C, arrowhead). Myh10 (D–F) and Myh14 (G–I) colocalized with actin filaments on both the apical (arrowhead) and basolateral membranes (arrow). NM2 isoforms appeared as punctate structures or short filaments on the membrane. Sections were mounted with vectashield containing DAPI to stain the nucleus (blue; C, F & I). Scale bar = 10 *μ*m.

NM2 is an actin‐associated motor protein that forms bipolar filaments along the actin cytoskeleton. To assess potential differences among the NM2 isoforms and their association with actin filaments in renal epithelial tubules, we performed immunofluorescence staining of mouse kidney sections using phalloidin to stain for actin filaments and one of the three NM2 isoforms. The NM2 isoforms (Myh9, 10, and 14) exhibited selective expression and localization along the apical and basolateral membranes of the tubules and did not localize with all the filamentous actin structures present in the tubules. In the areas where NM2 isoforms colocalized with the membrane‐associated actin filaments, they appeared as punctate structures as well as short filaments (Fig. 6B, E, H). The organization of the NM2 filaments on the membrane appeared as microdomain‐like structures that may be involved in localization, membrane targeting, and anchoring as well as endocytosis of ion channels, cotransporters, and other membrane‐associated proteins. Intracellular punctate staining for Myh10 and Myh14 isoforms was also observed in the renal epithelial cells (Fig. 6E, H).

## Discussion

Previous studies have shown that point mutations in the NM2A gene, *MYH9,* are associated with human diseases involving blood, eye, ear, and kidney disorders, a group of syndromes now collectively known as MYH9‐related diseases (Heath et al. 2001; Arrondel et al. 2002; Seri et al. 2003; Sekine et al. 2010). Some of the MYH9‐RD patients develop progressive proteinuria, glomerulosclerosis, and kidney failure (Pecci et al. 2008; Freedman et al. 2009; Oh et al. 2015). The variable occurrence of the kidney‐specific phenotype in MYH9‐RD patients is possibly due to the type of MYH9 mutation, along with variable compensation by the two other isoforms NM2B and NM2C (Wang et al. 2011; Zhang et al. 2012). Identifying NM2 as a critical player in regulating endocytosis opened up the possibility that in MYH9‐related diseases, defects in membrane trafficking pathways might be the central problem that leads to the disease pathology (Chandrasekar et al. 2013, 2014). In the mouse kidney, NM2A (Myh9) function has been postulated to maintain the structure of podocyte foot processes to ensure normal glomerular filtration of blood into the lumen of the nephron. However, the cellular function of NM2A in podocytes is not well understood and attempts to recapitulate loss of NM2 phenotype in mouse podocytes were not successful (Johnstone et al. 2013). We hypothesize that the endocytosis and receptor‐mediated transport in renal epithelial cells of the tubules might be the primary defect in *MYH9*‐disorders. Suboptimal levels of NM2A activity in the renal epithelial cells might lead to compromised trafficking of membrane‐associated proteins such as uromodulin that may initiate the progressive kidney disease in MYH9‐RD patients, resulting in secondary glomerular abnormalities. The first step to determine the role of NM2 in the renal tubules is to analyze the expression and localization pattern of the three NM2 isoform in adult mouse kidney. Although immunohistochemistry has been done looking at the distribution of NM2 in the kidneys of various species (Arrondel et al. 2002; Johnstone et al. 2011; Miura et al. 2013), we are unaware of any studies that have undertaken the task of colocalizing each of the NM2 isoforms with renal tubule‐specific markers. In this study, we utilized unique markers that are expressed within specific segments of the adult mouse renal tubules (megalin: proximal tubule; uromodulin: loop of Henle; calbindin D‐28K: distal tubule; aquaporin 2: collecting duct) and determined the expression and localization profile of NM2 isoforms. Interestingly, we found that each segment of the renal tubules predominately expressed only two of the three NM2 isoforms. Furthermore, the combination and the level of expression of the isoforms varied among segments.

Myh10 was the most ubiquitously expressed of the three isoforms and was found in all segments of the renal tubules (Fig. 1, 2, 3, 5) as well as the glomerulus (data not shown). In the renal tubules, Myh10 expression varied from high expression in the epithelial cells within the collecting duct (Fig. 5D–F) to less intense but specific subcellular locations like the cell–cell adhesion structures (tight junctions) in the distal tubule (Fig. 3E–H). The differences in expression and localization within each of these segments of the tubules suggest that Myh10 may play diverse roles throughout the kidney.

Our staining detected strong Myh9 expression in the thick ascending limb of the loop of Henle (Fig. 2A–C) and the glomerulus (Fig. 1D–F). Previous groups have provided evidence that Myh9 is expressed within the proximal tubule epithelia in humans and rats as well as in the loop of Henle in humans (Hosaka et al. 2009; Sekine et al. 2010). Uromodulin trafficking and release from the apical membrane in TAL cells is important for maintaining calcium homeostasis within the filtrate (Scolari et al. 2015; Devuyst et al. 2017) as well as regulating the immune response within the renal tubules (Saemann et al. 2005a,b). Individuals with mutations affecting uromodulin folding and subsequently its localization often have hereditary renal diseases that result in progressive tubulointerstitial damage and renal failure (Bleyer et al. 2003a,b). Because Myh9 has known functions in endocytosis (Chandrasekar et al. 2014) and vesicular trafficking (Miserey‐Lenkei et al. 2010), we postulate that defects in Myh9 function could lead to alterations in uromodulin localization leading to renal disease.

Myh14 was expressed throughout most segments of the renal tubules. Our assessment of Myh14 expression showed that the only segment where it was not expressed was the loop of Henle (Fig. 2G–I). In addition, Myh14 was not expressed in the glomerulus (data not shown). All other segments expressed Myh14 in combination with Myh10. Interestingly, in the distal tubule (Fig. 3) and the collecting duct (Fig. 4), Myh14 was expressed in the cells negative for Calbindin‐D28K, Aqp2, and Myh10. Staining for Atp6v1b1, a marker of intercalated cells in the collecting duct confirmed that the Myh14 expressing cells in distal tubules and collecting ducts are intercalated cell types. We are intrigued by the unique expression and localization of the Myh14 isoform in intercalated cells and not in principal cells, while Myh10 is expressed uniquely in principal cells and not intercalated cell types. Hence, within the distal tubules and collecting ducts Myh10 and Myh14 could serve as novel marker of principal and intercalated cell type, respectively.

Our studies were unable to determine how the NM2 isoforms localize relative to each other due to the lack of antibodies derived in species other than rabbit. As discussed earlier, NM2 isoform‐specific antibodies were generated using synthetic polypeptides constructed based on the unique c‐terminal (tail) 12–15 amino acid sequence among the isoforms. In some tubules it is clear that within the epithelial cells, localization pattern of each isoform is different, for example in the collecting duct, Myh10 is expressed in high levels on the Aqp2‐positive principal cells and localized to the membrane and the cytoplasm. However, Myh14 in the collecting duct was expressed only in the intercalated cells that do not express Aqp2 and instead expressed Atp6v1b1. In other segments such as the proximal tubule and loop of Henle, it is difficult to determine whether the two predominately expressed isoforms (Myh10 and Myh14; and Myh9 and Myh10, respectively) were localized to the same structures within the epithelial cells. All segments of the renal tubule can be further broken down into smaller regions that can be identified with staining for additional marker proteins. Further characterization of these regions is required to analyze the distribution of each of the NM2 isoforms within these subsegments. This will be essential to address isoform‐specific roles played by NM2 in various segments and subsegments within the renal tubules. Previous studies have shown that the three isoforms have distinct differences in ATP‐hydrolysis and actin‐binding properties (Billington et al. 2013). These differences have led to the discovery that each isoform has specific functions within various cells and tissue (Vicente‐Manzanares et al. 2011). However, there is redundancy between the functions of the NM2 isoforms in certain cell types and tissues (Wang et al. 2010, 2011). Our studies exploring the role of NM2A and NM2B in mammalian endocytosis indicate that both isoforms are equally involved in regulating endocytosis (Chandrasekar et al. 2014). Therefore, it is possible to observe compensatory mechanisms between the isoforms in vertebrate systems for certain cellular functions. The experimental data presented here is the first step toward our understanding of the role of NM2 in renal epithelium and possible isoform‐specific mechanisms involved in regulating the renal epithelial transport process. Further approaches like conditional genetic inactivation of NM2 isoforms only in the renal tubules and more specifically in distinct segments of the renal tubules will be necessary to determine the possible role of NM2 isoforms in regulating kidney function.

## Conflict of Interest

None declared.
